# Children With Biliary Atresia Have Substantial Morbidity in Early Childhood and a High Risk of Liver Transplantation

**DOI:** 10.1002/bdr2.70024

**Published:** 2026-02-08

**Authors:** Mads Damkjær, Joachim Tan, Maria Loane, Joanne Given, Elisa Ballardini, Clara Cavero‐Carbonell, Mika Gissler, Sue Jordan, Anna Pierini, Anke Rissmann, David Tucker, Ester Garne, Joan K. Morris

**Affiliations:** ^1^ Department of Paediatrics and Adolescent Medicine, Lillebaelt Hospital University Hospital of Southern Denmark Kolding Denmark; ^2^ Department of Regional Health Research University of Southern Denmark Kolding Denmark; ^3^ Population Health Research Institute, St George's University of London London UK; ^4^ NIHR GOSH Biomedical Research Centre, UCL Great Ormond Street Institute of Child Health London UK; ^5^ Faculty of Life & Health Sciences Ulster University Northern Ireland UK; ^6^ Neonatal Intensive Care Unit, University Hospital of Ferrara, IMER Registry (Emilia Romagna Registry of Birth Defects), Department of Medical Sciences, University of Ferrara Ferrara Italy; ^7^ Rare Diseases Research Unit, Foundation for the Promotion of Health and Biomedical Research in the Valencian Region Valencia Spain; ^8^ THL Finnish Institute for Health and Welfare, Department of Knowledge Brokers, Helsinki, Finland, Region Stockholm, Academic Primary Health Care Centre, Stockholm, Sweden; Karolinska Institutet, Department of Molecular Medicine and Surgery Stockholm Sweden; ^9^ Faculty of Health and Life Science Swansea University Swansea UK; ^10^ Unit of Epidemiology of Rare Diseases and Congenital Anomalies, Institute of Clinical Physiology, National Research Council Pisa Italy; ^11^ Malformation Monitoring Centre Saxony‐Anhalt, Medical Faculty Otto‐von‐Guericke‐University Magdeburg Magdeburg Germany; ^12^ Congenital Anomaly Registers & Information Service for Wales (CARIS), Public Health Knowledge & Research, Public Health Swansea UK

**Keywords:** biliary atresia, liver transplantation, morbidity

## Abstract

**Background:**

Biliary atresia is a rare but severe congenital anomaly associated with substantial morbidity and mortality in early childhood. Population‐based estimates of survival, surgical management, and liver transplantation across Europe remain limited. This study aimed to describe mortality and morbidity among children born with biliary atresia using multinational population‐based data.

**Methods:**

We investigated children diagnosed with biliary atresia across nine registries from five countries within the European surveillance of congenital anomalies network (EUROCAT), covering births from 1995 to 2014. The data were linked to hospital databases and adjusted for regional differences and follow‐up length.

**Results:**

Our cohort included 171 children, with an infant mortality rate of 12.3% (95% CI: 7.8–17.6) and a mortality rate before age five of 18.5% (95% CI: 10.7–27.7). Among these children, 151 had undergone surgery, including 133 who received the Kasai procedure by the age of 1 year at a median age of 57 days (95% CI: 51–62 days). By age five, 37% (adjusted percentage, 95% CI: 30–44) had undergone liver transplantation, with the median age at transplantation being 318 days (95% CI: 244–391 days). Median age at death in the first year was over 6 months and was not immediately after surgery.

**Conclusion:**

The high mortality and the substantial need for liver transplantation within the first year of life underline the severity of biliary atresia. This highlights the urgent need for further research into pregnancy exposures that may contribute to this rare but severe congenital anomaly to develop primary prevention strategies.

## 1 Introduction

Biliary atresia is a rare congenital anomaly which affects around 5 of 100,000 livebirths worldwide, with international variations (Jimenez‐Rivera et al. 2013). Prevalence seems to be higher in East Asia than in Europe and North America and there is some evidence of seasonality in prevalence (Jimenez‐Rivera et al. 2013). The etiology is unknown, but fetal exposure to maternal infections or toxins may explain some of the cases (Fawaz et al. 2017; Mysore et al. 2019; Tiao et al. 2008). As these fetal exposures may be preventable, biliary atresia may be a preventable anomaly (Turmelle and Shepherd 2008).

The main symptom of biliary atresia is prolonged jaundice with elevated conjugated bilirubin and therefore the anomaly is asymptomatic at birth but can be diagnosed later in the neonatal period (Islek and Tumgor 2022; Jimenez‐Rivera et al. 2013). The recommended treatment is to perform the Kasai surgical procedure, involving removal of the defective bile ducts and connection of a segment of small intestine to the liver to allow bile drainage as early as possible to reduce the need for liver transplantation (Kelley‐Quon et al. 2022).

The EUROlinkCAT project aimed to analyze the morbidity of live born children with major congenital anomalies by linking data from the population‐based EUROCAT registries of congenital anomalies to hospital databases, to obtain information on length of hospital stays and surgical procedures (Morris et al. 2021). Children with biliary atresia had a median length of stay in hospital in the first year of life at 53.8 days (95% CI: 46.8–60.8) and a median number of any surgical procedure at 3.6 (95% CI: 2.6–4.7) during the first 5 years, indicating a high burden of disease in early childhood (Garne, Tan, et al. 2023).

The aim of this study is to report mortality and morbidity for children born with biliary atresia with a focus on Kasai surgery and liver transplantation and to compare the burden of hospitalisations to children without congenital anomalies and to all children with congenital anomalies.

## 2 Methods

This is a European, population‐based data‐linkage cohort study using routinely collected data from hospital discharge databases. The study includes data from nine EUROCAT registries (national and regional) in five countries (Denmark, Finland, Italy, Spain, and UK, see Table 1).

**TABLE 1:** Registries and children included in the study.

EUROCAT registry	Birth years included	Number of children		Number of children with biliary atresia
		Reference children	EUROCAT children	
Denmark, Funen	1995–2014	100,748	2423	5
Finland	1997–2014	911,679	38,324	69
Italy, Emilia‐Romagna	2008–2014	223,995	5381	16
Italy, Tuscany	2005–2014	23,503	4225	2
Spain, Valencian Region	2010–2014	168,563	4260	19
UK, Wales	1998–2014	531,784	17,448	41
UK, East Midlands & South Yorkshire	2003–2012	Data for reference children not available	11,278	19^a^
UK, Thames Valley	2005–2013		3845	
UK, Wessex	2004–2014		4320	
Total		1,960,272	91,504	171

^a^ Due to small number suppression all English registries are not reported individually.

We included all children born alive between 1995 to 2004 in the geographic areas covered by the EUROCAT registries and diagnosed with biliary atresia. All children in the study were followed from birth to their 5th birthday or to the end of 2015, whichever came first; thus, all children had at least 1 year of follow‐up.

Data on hospitalisations and surgical procedures were obtained by electronic linkage to the hospital databases used in the regions and countries. Further details of the linkage methods used in the EUROlinkCAT study have been published earlier (Morris et al. 2021; Urhoj et al. 2022). For six of the nine registries, the hospital databases covered hospital stays in the whole country. For Wales, hospital stays in England (funded by the Welsh NHS) were included. For the Valencian Region in Spain and Emilia Romagna in Italy, the hospital databases covered the same region as the EUROCAT registry. Data on hospitalization and surgery for reference children and children with any congenital anomaly for comparison were obtained from previous EUROlinkCAT publications (Garne, Loane, et al. 2023; Urhoj et al. 2022).

Surgical procedures were coded according to the coding systems used in the national health systems. Italy and Spain used ICD‐9‐CM (the International Classification of Diseases, 9th revision—Clinical Modification) for the study period; the UK used OPCS‐4 (Classification of Interventions and Procedures), and Finland and Denmark used national adaptations of NCSP (NOMESCO Classification of Surgical Procedures). Three pediatricians from two European countries independently reviewed all the codes from the extensive lists of surgery and procedure codes from the five countries with the three different coding systems and reached consensus on which codes defined surgery. The surgery variables “any surgery”, “intestinal surgery”, “Kasai surgery” and “liver transplantation” were defined in each of the coding systems.

## 3 Statistics

Due to privacy regulations, direct sharing of individual case data among registries was not possible. To overcome this, a common data model was adopted across all participating registries, allowing for the application of a standardized analysis script (Stata version 13) by each registry independently. The aggregated results and tables generated from these analyses were then combined using meta‐analytic techniques to produce estimates that reflect the European context.

The analysis was stratified by age groups (less than 1 year, 1–4 years, and 0–4 years) and adjusted for both the birth region/country and the lengths of follow‐up (which varied across registries). For infants, the proportion undergoing any surgical procedure was calculated against the total live births. Due to incomplete follow‐up in some registries, Kaplan–Meier estimates were used to estimate the proportion of children undergoing surgery. The overall surgery rates were calculated using the METAN command in Stata, employing the Freeman‐Tukey double‐arcsine transformation and inverse‐variance method for back‐transformation of pooled effects. Registry‐reported medians and interquartile ranges for hospital stay durations, surgical procedure counts, and ages at first surgery were combined using meta‐analytic methods detailed in prior publications.

## 4 Results

The analysis included 171 children diagnosed with biliary atresia and born in 1995–2014 in nine regions across Western Europe (Table 1). There were 21 deaths in the first year which gives an infant mortality of 12.3% (95% CI: 7.8–17.6). An additional eight children died before age 5 years, with a mortality rate up to age 5 of 18.5% (95% CI: 10.7–27.7) after adjusting for many children not being followed up for the full 5 years.

A total of 151 children had a code for any surgery, including the specific code for the Kasai procedure, performed by the first year of age. The Kasai procedure was performed in 133 children (78% of total) before 5 years of age with a median age at surgery of 57 days (95% CI: 51–62 days) (Table 2). The study also analyzed 30‐day mortality after surgery in the neonatal period. Nineteen children had a code for any intestinal surgery before the age of 28 days (including the code for the Kasai procedure), and all were alive 30 days after their surgery. Only 10 of these children had the specific code for the Kasai procedure. Median age at death in the first year was over 6 months and was not immediately after surgery.

**TABLE 2:** Surgical procedures in children with biliary atresia, based on the codes for surgery in the hospital databases.

	Number	% of total (95% CI)	Median age in days at first surgery (95% CI)	Median number of surgeries for children with any code for surgery (95% CI)
Children with biliary atresia	171	100%		
Children with any code for surgery < 1 year	151	88% (82–93)		2.1 (1.4–2.9)
Children with a specific code for Kasai surgery < 5 years	133	78% (71–84)	57 days (51–62)	1.0 (1.0–1.0)
Children with a code for liver transplantation < 5 years	52	37% (30–44)	318 days (244–391)	1.2 (1.0–1.7)
Survivors at 1 years without any codes for surgery	1	0.6% (0.0–3.2)		
Children alive at 1 year	148	88% (82–92)		

For the children who were followed up to age 5 years, 52 had a code for liver transplantation in the hospital databases (adjusted percentage was 37% (95% CI: 30–44) to allow for loss to follow‐up). The median age at first liver transplantation was 318 days (95% CI: 244–391 days). The median number of liver transplantations for these 52 children was 1.2 (95% CI: 1.0–1.7).

**FIGURE 1:**
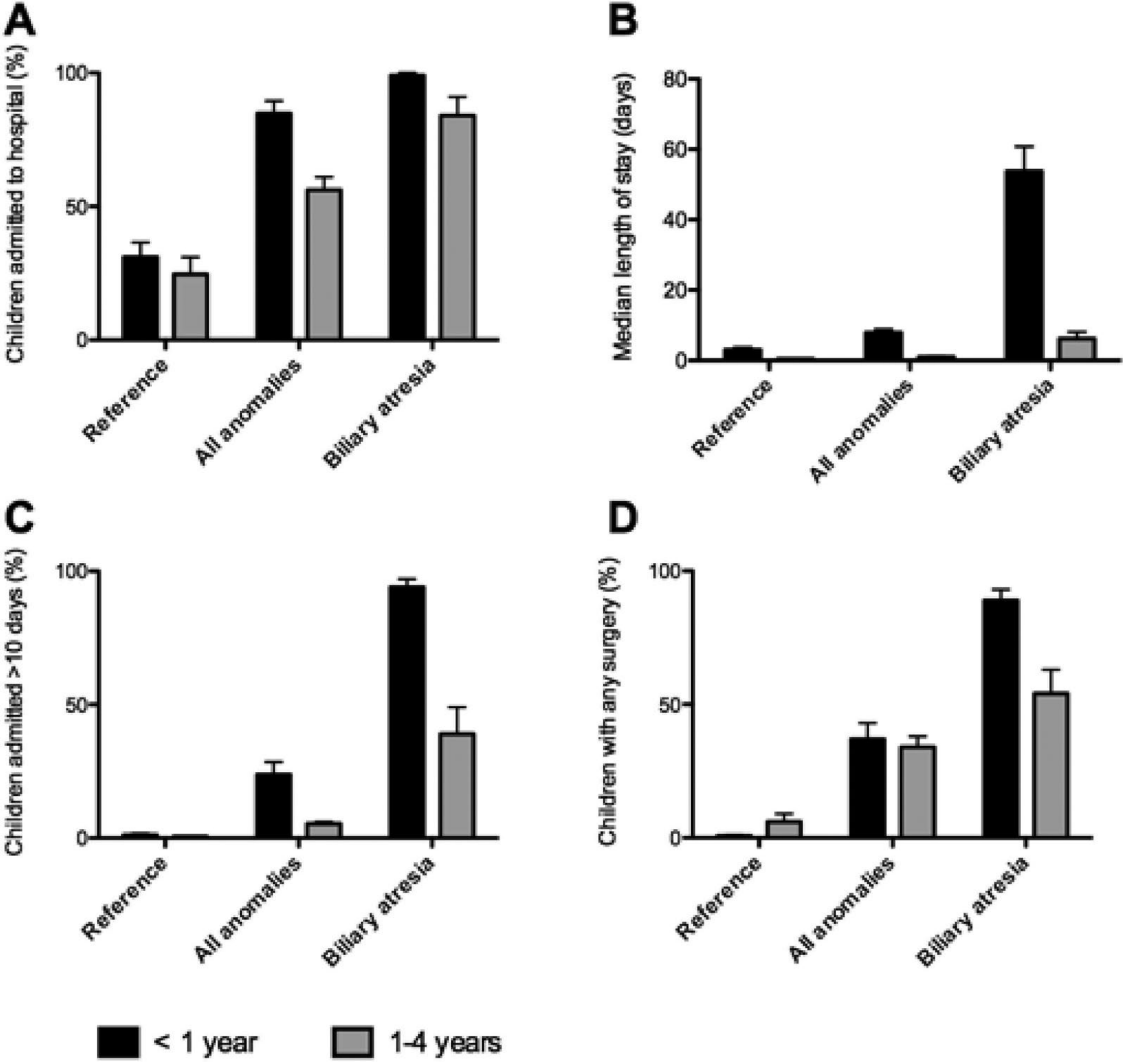
The burden of hospitalisations in children with biliary atresia compared to all children with congenital anomalies and to children without congenital anomalies (Reference). The data, covering nine population‐based registries in five countries from 1995 to 2014, illustrate the percentage of children admitted to the hospital (A), the median length of hospital stay in days (B), the percentage of term born children with a hospital admission extending beyond 10 days (C), and the percentage of children undergoing any surgery (D).

The burden of hospitalization in the first year of life for children with biliary atresia was compared to children with any congenital anomaly and to children without congenital anomalies in Figure 1. The morbidity –measured as proportion hospitalized, median length of stay for all children, proportion of term born children with at least one hospital stay of more than 10 days, and proportion having any surgery—was considerably higher for children with biliary atresia than for the children in the two other groups.

## 5 Discussion

Our results showed significant mortality in early childhood for children born with biliary atresia, as evidenced by an infant mortality at 12.3% (95% CI: 7.8–17.6) and a five‐year mortality of 18.4% (95% CI: 10.7–27.7). Our results also showed a high burden of hospitalisations in early childhood with long hospital stays, surgical procedures in infancy and the need for liver transplantation.

The median age at Kasai surgery was 57 days (95% CI: 51–62 days) in our population of children born in 1995–2014. An American study across 46 children's hospitals also found the median age at Kasai surgery to be 57 days (interquartile range: 42–72 days) and 45% of the children were more than 60 days old (Apfeld et al. 2021). This may reflect the difficulties in diagnosing biliary atresia early.

We found that about one out of three children had a liver transplantation before the age of 5 years, with most transplantations taking place before age 1 year. Evidence suggests that more than half of the children with biliary atresia will undergo liver transplantation before the age of 2 years (Arnon et al. 2016; Kastenberg et al. 2023). Our estimate of the median age at transplantation is therefore consistent with previous studies. The proportion of children needing liver transplantation may be underestimated, as not all children who need a transplant will receive one in time. Other reasons for underestimating the proportion of children undergoing liver transplantation in our study may be incomplete recording of transplantation procedures in the hospital databases, transplantation taking place in specialist centres outside the hospital and registry catchment area, and that affected children and their families may also move outside the study areas within the first 5 years after birth.

Parents of children with biliary atresia may also worry about other long‐term outcomes than those directly related to liver function, as studies have shown a small association between exposure to anesthesia and surgery in young children and later academic performance (Glatz et al. 2017; Rosenblatt et al. 2019). A study including 41 children with biliary atresia and a median age at 11 years at follow‐up showed that 26% of the children had received special education and only 25% had normal motor outcomes (Rodijk et al. 2020). Total IQ was also lower compared to the background population (mean ± SD: 91 ± 18 vs. 100 ± 15; *p* < 0.01). Therefore, children with biliary atresia need specialized follow‐up during childhood by a multidisciplinary team including experts in both pediatric hepatology and pediatric neurology.

An international guideline with recommendations for general screening of infants with extended jaundice was published in 2017 (Fawaz et al. 2017). The guideline recommends measurements of conjugated bilirubin for all infants with visible jaundice at 2 weeks of age for early diagnosis of biliary atresia. Studies from Utah and Texas have shown that their screening programmes have a very high sensitivity in diagnosing biliary atresia within the first weeks after birth (Harpavat et al. 2020; Kastenberg et al. 2023) and reduced the mean age at the Kasai procedure from 56 days to 36 days (Harpavat et al. 2020). However, most of the children in our study were born before such screening was recommended. Our study only included data on children born up to 2014. However, as noted by (Wehrman et al. 2019), ‘the management of BA has not changed significantly in the past decade,’ suggesting that significant improvements in outcomes for children born more recently are unlikely to have occurred. This is also well in line with a study from France which found that overall 5‐year survival did not change from 1997 to 2009 (Chardot et al. 2013).

We found that nearly 1 out of 5 children born with biliary atresia died before the age of 5 years. Our results also confirm a high morbidity in infancy and early childhood for these children. In this European population, 1 of 3 children born with biliary atresia underwent liver transplantation before age 5 years, with the majority in the first year. We found that the median age at death in the first year was over 6 months and was not immediately after surgery. This suggests that these infants die because of liver failure complications due to their severe anomaly and not around the time of diagnosis. This is important information for parental counseling and care planning after a diagnosis. It also shows that more progress is needed for earlier identification of children with biliary atresia, thus allowing for earlier intervention with the Kasai procedure and, in turn, the potential to decrease the need for subsequent liver transplantation and long‐term immunosuppressant therapy (which, in itself, is an appreciable treatment burden). More research on pregnancy exposures related to the occurrence of biliary atresia is highly recommended to find methods of primary prevention of this rare and very severe congenital anomaly.

## Funding

This project has received funding from the European Union's Horizon 2020 research and innovation programme under grant agreement No. 733001. The funders had no role in the study.

## Ethics Statement

All EUROCAT registries obtained ethical and other permissions for the data linkage according to their national legislations. University of Ulster obtained ethics permission for the Central Results Repository on 15 September 2017 (Institute of Nursing and Health Research Ethics Filter Committee, number FCNUR‐17‐000).

## Conflicts of Interest

The authors declare no conflicts of interest.

## Data Availability

The data that support the findings of this study are available from the participating registries of congenital anomalies, but restrictions apply to the availability of these data, which were used under license for the current study. These data are available for scientifically valid requests and with permission of the participating registries of congenital anomalies. To apply for the data please contact the corresponding author.
